# Association between risk of obstructive sleep apnea severity and risk of severe COVID-19 symptoms: insights from salivary and serum cytokines

**DOI:** 10.3389/fpubh.2024.1348441

**Published:** 2024-02-21

**Authors:** Yen Dinh, Abdullah Alawady, Hesham Alhazmi, Khaled Altabtbaei, Marcelo Freire, Mohammad Alghounaim, Sriraman Devarajan, Fahd Al Mulla, Saadoun Bin-Hassan, Hend Alqaderi

**Affiliations:** ^1^Harvard School of Dental Medicine, Boston, MA, United States; ^2^Ministry of Health, Kuwait City, Kuwait; ^3^Faculty of Dentistry, Kuwait University, Kuwait City, Kuwait; ^4^Department of Preventive Dentistry, Faculty of Dentistry, Division of Pediatric Dentistry, Umm Al-Qura University, Mekkah, Saudi Arabia; ^5^School of Dentistry, Faculty of Medicine and Dentistry, University of Alberta, Edmonton, AB, Canada; ^6^Dasman Diabetes Institute, Dasman, Kuwait; ^7^Department of Genomic Medicine and Infectious Diseases, J. Craig Venter Institute, La Jolla, CA, United States; ^8^Division of Infectious Diseases and Global Public Health Department of Medicine, University of California San Diego, La Jolla, CA, United States; ^9^Tufts University School of Dental Medicine, Boston, MA, United States

**Keywords:** COVID-19, SARS-CoV-2, obstructive sleep apnea, IL-6, cytokines

## Abstract

**Objectives:**

Obstructive sleep apnea (OSA) can adversely affect the immune response through clinical factors such as hypoxia, inflammation, and sleep disturbance. Since SARS-CoV-2 heavily relies on local and systemic host immune responses, this study aims to examine the links between the severity of OSA risk, cytokine levels, and the severity of symptoms associated with SARS-CoV-2 infection.

**Methods:**

Saliva and blood samples from 50 COVID-19 patients and 30 non-infected hospital staff members were collected. Using Luminex multiplex analysis, 65 blood and salivary cytokines were examined from the collected samples. Ordinal logistic regression analysis was utilized to examine the association between the self-reported risk of OSA, assessed through the STOP-Bang questionnaire, and the likelihood of experiencing severe symptoms of COVID-19. Mann–Whitney test was then performed to compare the cytokine levels between individuals with moderate to severe risk of OSA to those with a mild risk of OSA.

**Results:**

Ordinal logistic regression analysis revealed that individuals with a moderate to severe risk of OSA were 7.60 times more likely to experience more severe symptoms of COVID-19 compared to those with a mild risk of OSA (OR = 7.60, 95%CI: 3.03, 19.06, *p* < 0.001). Moreover, among COVID-19-positive patients with a moderate to severe risk of OSA, there was a statistically significant negative correlation with serum IL-6 (*p* < 0.05), Eotaxin (CCL11) (*p* = 0.04), and salivary MIP-3α/CCL20 (*p* = 0.04). In contrast, individuals without COVID-19 who had a moderate to severe risk of OSA exhibited a significant positive correlation with serum IL-6 (*p* = 0.04).

**Conclusion:**

Individuals with moderate to severe risk of OSA were more likely to experience severe COVID-19 symptoms than those with mild risk for OSA. Additional analysis from the present studies revealed distinct patterns of oral and systemic immune responses between individuals with mild and moderate to severe risk of OSA. Findings from the present study underscores the importance of early detection and management of OSA to improve clinical outcomes, particularly when faced with the subsequent superimposed infection such as COVID-19.

## Introduction

Obstructive sleep apnea (OSA) affects approximately one billion individuals globally and is characterized by periodic interruptions in breathing during sleep, either partially or completely (1, 2). Individuals with OSA face an elevated risk of developing cardiovascular disease, hypertension, and various other chronic health issues (2, 3). The potential link between OSA and COVID-19 has been of particular interest due to the respiratory nature of both conditions, their overlapping comorbidities, and notable risk factors, particularly obesity (4).

Persistent, episodic collapse of airway during sleep results in chronic intermittent hypoxia, a state of decreased oxygen saturation and increased carbon dioxide arterial blood partial pressure (5). Each time this occurs, the process of desaturation and reoxygenation results in oxidative stress and a generation of reactive oxygen species (ROS) (5). Ultimately, chronic intermittent hypoxia can result in immune dysfunction both locally and systemically, predisposing individuals with OSA to developing subsequent superimposed infections (6, 7).

In recent years, the SARS-CoV-2 and the successive variants has had a significant, widespread impact worldwide. The World Health Organization (WHO) reported over 761 million cases and 6.8 million deaths to date (8). In particular, COVID-19 is caused by SARS-CoV-2 and primarily affects the respiratory system. Symptoms range from asymptomatic to severe Acute Respiratory Distress Syndrome (ARDS), requiring hospitalization and potentially leading to mortality (9). Multiple risk factors, including obesity, age, diabetes mellitus (DM), and cardiovascular disease, have been linked with an increase in severity of COVID-19 symptoms (10).

Previous studies illustrated the presence of similar risk factors in individuals with OSA, hinting at a possible connection between OSA and the severity of COVID-19 symptoms (11, 12). Earlier investigations also revealed that individuals who had both OSA and contracted COVID-19 faced an elevated risk of hospitalization, mechanical ventilation, admission to intensive care units (ICUs), and mortality when compared to COVID-19 patients with OSA (4, 13, 14). Despite these notable findings, there remains a substantial gap in the existing body of literature, prompting the current study to focus on investigating the relationship between risk of OSA, the severity of COVID-19 symptoms, and their association with relevant serum and salivary biomarkers.

## Methods

The present study was conducted in collaboration with the Dasman Diabetes Institute in Kuwait, the J Craig Venter Institute (JCVI), the Ministry of Health in Kuwait, and the University of Alberta. Ethics approval was granted by the Institution Review Boards (IRB) of JCVI, the Ministry of Health in Kuwait, and the University of Alberta. All enrolled participants provided their informed consent for participation in the study, with the respective ethics approval references as follows: Kuwait Ministry of Health #2020/1462, the University of Alberta #Pro00125245, and JVCI, which received an exemption due to the secondary analysis of de-identified samples.

### Study design

The present study employed a convenience sampling approach, enrolling patients from AlFarwaniyah, Jaber Al Ahmed, and Kuwait Field hospitals in Kuwait between 24 July and 4 September 2020. Individuals who tested positive for SARS-CoV-2 by RT-PCR (*n* = 50) provided consent and underwent nasopharyngeal swab collection. For the control group, nasopharyngeal swabs were obtained from hospital staff members (*n* = 30) who had no contact with COVID-19 case(s).

Using a Qualtrics questionnaire on an iPad, comprehensive demographic and clinical data were gathered from the participants. This included details about their medical history, medication use, smoking habits, sleep patterns, weight, height, waist circumference, neck circumference, blood type, respiratory rate, COVID-19 symptoms, and, for those using supplemental oxygen, the specific liter amount.

### Saliva collection

In preparation for saliva collection, 15 mL plastic centrifuge tubes were pre-labeled with the date and subject number, along with a clear demarcation at the 4 mL level. The saliva collection tubes were then placed on ice. Before proceeding with sample collection, a hospital nurse provided detailed instructions and demonstrated to the participants the process of saliva collection, including how to use the parafilm to stimulate saliva production and how to collect the saliva in the pre-marked tubes. The participants were directed to swish a sip of water in their mouth, swallow it, and then chew on a piece of parafilm. When saliva formed, the participants used their tongue to push the saliva into the pre-marked tube, which was placed back in an ice-filled cup. Saliva collection continued until it reached the 4 mL mark, accounting for the meniscus. After completing the task, participants notified the nurse, who then sealed and sanitized the tube before storing it in an ice-filled collection rack and disposing of any unused materials.

### Blood collection

All serum samples were collected in 7.5 mL BD Vacutainer Serum tubes with clot activator using standardized venipuncture techniques.

### Sample processing

The samples were packed in containers with dry ice and transferred to the Jaber Alahmad Hospital laboratory. The samples were received and processed within the same day, ensuring a maximum of 3 h elapsed between collection and processing. Salivary samples were centrifuged at 2000xg for 5 min. The separated supernatant and pellets were transferred into distinct tubes. Serum samples were centrifuged at 2000xg for 10 min after a 30-min interval of equilibration at room temperature on vertical racks. All processed samples were stored at −80°C. During samples transfer from the laboratory to JCVI, the samples were placed on dry ice along with a specialized monitoring apparatus to guarantee their sustained frozen condition throughout the transportation process.

### Cytokine abundance measurements

The serum analysis was conducted with the Luminex 200 system (Luminex Corporation, Austin, Texas, United States) using the Immune Monitoring 65-Plex Human ProcartaPlex Panel (Cat# EPX650-16500-901; ThermoFisher Scientific, Vienna, Austria) following the manufacturer’s instructions. This comprehensive kit assessed immune function by examining 65 protein targets in a single well, encompassing cytokines, chemokines, growth factors/regulators, and soluble receptors. To establish a standard curve, the provided standard was diluted fourfold, and both high and low controls were incorporated into the analysis.

### Outcome variable

#### COVID status

COVID-19 severity was categorized into four distinct groups as follows: (1) Mild symptoms, which included individuals who were hospitalized without the need for oxygen therapy (*n* = 11); (2) Moderate symptoms, involving hospitalized patients requiring low-flow oxygen support (<10 L/min) (*n* = 28); (3) Severe symptoms, comprising of hospitalized patients who need high-flow oxygen support (>10 L/min) (*n* = 11); and (4) the control group, composed of hospital administrative staff members who had no contact with COVID-19 patients (*n* = 30). The control group underwent daily visual triage by the hospital, including temperature and symptom checks, although they did not undergo PCR testing nor have a confirmed negative PCR test. The control group was age-and sex-matched with the COVID-19 subjects. Two variables were generated for analysis, a binary variable and a categorical variable. Stratification of the participants into COVID-19 vs. no COVID-19 groups yielded a binary variable for the descriptive statistics. For the ordinal logistic regression analysis, a categorical variable was generated after grouping participants into one of four categories: (1) control (non-infected); (2) mild symptoms; (3) moderate symptoms; and (4) severe symptoms.

### Exposure variable and covariates

#### Obstructive sleep apnea

The risk of OSA was evaluated using the STOP–Bang questionnaire, a well-validated screening tool known for its high sensitivity of 96% and a negative predictive value of 90% (15). Participants were asked the following questions: (1) Do you snore loudly (loud enough to be heard through closed doors or your bed-partner elbows you for snoring at night)? (2) Do you often feel tired, fatigued, or sleepy during the daytime (such as falling asleep during driving or talking to someone)? (3) Has anyone observed you stop breathing or choking/gasping during your sleep? (16). Participants received one point for each affirmative response to the aforementioned questions. An extra point was included if they were male, aged 50 or older, had a body mass index (BMI) exceeding 35 kg/m (2), possessed a neck circumference exceeding 40 cm, or had a history of high blood pressure. Participants who accumulated three or fewer points were classified as having a low risk of OSA, whereas those with more than three points were categorized as having a moderate to severe risk of OSA (16).

#### Body measurements

Measurements for height, weight, waist, and neck circumferences were collected using the Qualtrics software on an iPad. Weight was assessed using a standard scale, while height was determined with the integrated stadiometer. Waist circumference was measured using a paper anthropometry tape, specifically at the midpoint between the bottom of the rib cage and the top of the iliac crest. These measurements were taken for each participant during minimal respiration and recorded to the nearest 0.1 cm. For neck circumference measurement, participants were advised to stand in a relaxed posture with their head held upright, looking straight ahead. The measurement was taken just above the midpoint of the participant’s neck, usually below the thyroid cartilage (commonly known as the Adam’s apple), at the end of a normal expiration. All measurements were recorded to the nearest millimeter.

### Statistical analysis

All statistical analyses were carried out utilizing STATA 17 software, where a significance threshold of 0.05 was set. To create demographic data represented in Tables 1, 2, categorical variables were examined using chi-square tests, resulting in counts and percentages. To explore the connection between COVID-19 status and OSA risk, an ordinal logistic regression analysis was conducted after adjusting for gender and age. Moreover, an analysis of sixty-five serum and salivary biomarkers was performed. For each of these biomarkers, calculations were conducted to determine both the median and interquartile range (IQR) with a distinction between individuals with a mild risk of OSA and those with a moderate to severe risk. Following this, a two-sample Mann–Whitney test was employed to assess the statistical significance of differences between these two groups for each respective biomarker.

**Table 1:** Descriptive summary of sample characteristics stratified by risk of obstructive sleep apnea.

Characteristics	Mild risk of obstructive sleep apnea *N* = 46 (%)	Moderate/severe risk of obstructive sleep apnea *N* = 34 (%)	*p*-values
Age ≤ 50 years	40/46 (87)	11/33 (32.3)	<0.001
Age > 50 years	6/46 (13)	23/33 (68.7)	
Female	24/46 (52.1)	12/34 (35.3)	0.13
Male	22/46 (47.9)	22/34 (64.7)	
Diabetic	7/46 (15.2)	16/34 (47)	0.002
Non-diabetic	39/46 (84.8)	18/34 (53)	
Heart disease	1/46 (2.2)	7/34 (20.6)	0.007
No heart disease	45/46 (97.8)	27/34 (79.4)	
Hypertension	2/46 (4.4)	20/34 (58.8)	0.000
No hypertension	44/46 (95.6)	14/34 (41.2)	
BMI ≤ 35	39/46 (84.8)	18/34 (52.9)	0.002
BMI >35	7/46 (15.2)	16/33 (47.1)	
Smoker	15/43 (34.9)	3/34 (8.8)	0.007
Non-Smoker	28/43 (65.1)	31/34 (91.2)	
Asthma	2/45 (4.4)	3/34 (8.8)	0.43
No asthma	43/45 (95.6)	31/34 (91.2)	
Emphysema	6/46 (13.1)	0/34 (0)	0.03
No emphysema	40/46 (86.9)	34/34 (100)	
Male waist circumference ≤ 94	3/22 (13.6)	2/22 (9.1)	0.64
Male waist circumference > 94	19/22 (86.4)	20/22 (90.1)	
Female waist circumference ≤ 80	6/24 (25)	0/12 (0)	0.06
Female waist circumference > 80	18/24 (75)	12/12 (100)	
Male neck circumference ≤ 43	20/22 (90.9)	19/22 (86.4)	0.64
Male neck circumference > 43	2/22 (9.1)	3/22 (13.6)	
Female neck circumference ≤ 41	21/24 (87.5)	10/12 (83.3)	0.73
Female neck circumference > 41	3/24 (12.5)	2/12 (16.7)	

*p*-values were determined using chi-square tests. Waist circumference cut off was based on the International Diabetes Federation. Cut off for males was 94 cm, while for females it was 80 cm. Neck circumference cut off was based on the WHO guidelines. Cut off for males was 43 cm, while for females it was 41 cm.

**Table 2:** Descriptive summary of COVID-19 symptoms stratified by risk of obstructive sleep apnea.

Characteristics	Mild risk of obstructive sleep apnea *N* = 46 (%)	Moderate/severe risk of obstructive sleep apnea *N* = 34 (%)	*p*-values
Mild COVID-19 symptoms	7/19 (36.84)	4/31 (12.90)	0.56
Moderate/severe COVID-19 symptoms	12/19 (63.16)	27/31 (87.10)	
Shortness of breath	11/19 (57.9)	21/31 (67.7)	0.48
No shortness of breath	8/19 (42.1)	10/31 (32.3)	
respiratory distress <=20/min	5/46 (10.9)	9/34 (26.5)	0.07
Respiratory distress >20/min	41/46 (89.1)	25/34 (73.5)	
Chest pain	9/45 (20)	6/34 (17.7)	0.79
No chest pain	36/45 (80)	28/34 (82.3)	
Sputum production	8/19 (42.1)	15/31 (48.4)	0.67
No sputum production	11/19 (57.9)	16/31 (51.6)	
Cough	17/19 (89.5)	25/31 (80.6)	0.41
No cough	2/19 (10.5)	6/31 (19.3)	
Congestion	4/19 (21.1)	4/31 (12.9)	0.45
No congestion	15/19 (78.9)	27/31 (87.1)	
Sore throat	6/19 (31.6)	8/31 (25.8)	0.66
No sore throat	13/19 (68.4)	23/31 (74.2)	

Symptom severity was based on oxygen supplementation requirements. Subjects were placed in the Mild symptoms category if they were hospitalized without the need for oxygen therapy. Moderate if they required less than 10 L/min and severe if they required > 10 L/min of 100% O_2_ supplementation Subjects were placed in the severe symptoms category if they required > 10 L/min of 100% O_2_ supplementation. Respiratory distress cut off was based on 20 breaths/min.

## Results

Among the 80 participants of this study, 42.5% of individuals had moderate to severe risk of OSA while 57.5% had mild risk of OSA. For those with moderate to severe risk of OSA, 64.7% were male, 68.7% were > 50 years old, 47.1% had a BMI >35 kg/m2, and 91.2% were smokers (Table 1).

87% of individuals who experienced moderate and severe COVID-19 symptoms had a moderate to severe risk of OSA (Table 2).

COVID-19 positive individuals with a moderate to severe risk of OSA exhibited lower serum IL-6 levels (median 4, IQR 28) compared to individuals with a mild risk of OSA (median 21, IQR 38, *p* < 0.05). In contrast, individuals without a COVID-19 but who have a moderate to severe risk of OSA displayed higher serum IL-6 levels (median 14, IQR 25.5) than those with a mild risk of OSA (median-8, IQR 5, *p* < 0.05) (Table 3).

**Table 3:** Descriptive summary of statistically significant differences between serum and salivary biomarker levels stratified by COVID-19 status and risk of OSA.

Biomarkers:		No COVID		*p*-values	COVID		*p*-values
		Mild risk of OSA Median (IQR)	Moderate/Severe risk of OSA Median (IQR)		Mild risk of OSA Median (IQR)	Moderate/Severe risk of OSA Median (IQR)	
Serum (pg/ml)	IL-6	−8 (5)	14 (25.5)	0.04	21 (38)	4 (28)	0.04
	MIP-3α/CCL20	0 (4)	−1 (1)	0.15	3 (7)	4 (7)	0.42
	Eotaxin-1/CCL11	2056 (1503.5)	1,124 (1017)	0.32	1933 (1210)	1332.5 (1178)	0.04
Saliva (pg/ml)	IL-6	0.067 (0.248)	0.015 (0.072)	0.33	0.014 (0.154)	0.007 (0.366)	0.95
	MIP-3α/CCL20	0.276 (0.404)	0.08 (0.196)	0.12	0.293 (0.688)	0.205 (0.286)	0.04
	Eotaxin-1/CCL11	0.106 (0.235)	0.106 (0.059)	0.70	0.117 (0.161)	0.146 (0.198)	0.94

Mann–Whitney test was used to determine the *p*-values. IQR, interquartile range.

Among individuals who tested positive for COVID−19, those with a moderate to severe risk of OSA exhibited lower levels of salivary MIP-3α/CCL20 (Median 0.205, IQR 0.286) compared to those with a mild OSA risk (Median 0.293, IQR 0.688, *p* < 0.05). Furthermore, in COVID-19-positive individuals with a moderate to severe OSA risk, lower levels of serum Eotaxin-1/CCL11 (median 1332.5, IQR 1178) were observed in contrast to those with a mild OSA risk (median 1933, IQR 1210, *p* < 0.05) (Table 3).

Individuals with a moderate to severe risk of OSA exhibit a 7.60-fold increased likelihood of falling within higher categories of COVID-19 symptom severity (OR: 7.60, 95%CI: 3.03–19.06, *p* < 0.001). Specifically, the probability of transitioning from lower (control or mild) to higher (mild to moderate, moderate to severe) COVID-19 symptom categories is 7.60 times higher among individuals with a moderate to severe risk of OSA compared to those with a mild risk (Table 4).

**Table 4:** Ordinal logistic regression analysis of the association between COVID-19 status and risk of OSA.

Covariate	Odds ratio	95% confidence interval	*p*-values
Sleep apnea	7.60	3.03–19.06	<0.001
Adjusted sleep apnea*	5.85	2.38–14.41	<0.001

*The adjusted sleep apnea variable was determined using the STOP-Bang model while excluding BMI.

Sensitivity analysis was conducted using the same sleep apnea variable while excluding BMI from the variable. Individuals at a moderate/severe risk of OSA still exhibited a 5.85 fold increased likelihood of falling within the higher categories of COVID-19 symptom severity (OR: 5.85, 95%CI: 2.38–14.41, *p* < 0.001).

## Discussion

The present study investigated the association between the risk of OSA and the likelihood of individuals experiencing heightened COVID-19 symptoms, while also examining the associated inflammatory patterns of the disease versus the non-diseased state. We demonstrated that individuals with moderate to severe risk for OSA were more likely to experience more severe symptoms than individuals with mild risk for OSA. Additionally, the sensitivity analysis revealed that our results remained statistically significant despite BMI being excluded from the sleep apnea variable, suggesting that the association between risk of OSA and COVID-19 symptom severity is not solely driven by obesity, rather by the combination of the different components of the STOP-Bang model (gender, age, BMI, hypertension, snoring, gasping for air during sleep, feeling sleepy during the daytime, and neck circumference).

Furthermore, individuals with a moderate to severe risk of OSA, in the absence of concurrent COVID-19, exhibited elevated levels of serum IL-6 in contrast to those with a mild risk of OSA. Conversely, individuals at moderate to severe risk of OSA with concurrent COVID-19 showed reduced levels of serum IL-6, Eotaxin-1/CCL11, and salivary MIP-3α/CCL20 compared to those with mild OSA risk and concurrent COVID-19. Since it is widely recognized that OSA can compromise the immune system, rendering individuals with preexisting OSA more susceptible to experiencing severe symptoms due to their weakened immune response against the virus.

The increased serum levels of IL-6 in patients with a moderate to severe risk of OSA, without concurrent COVID-19, aligns with the findings made by Imani et al. (17). Specifically, IL-6 plays various roles in inflammation and the immune system, including the stimulation of immunoglobulin secretion and the initiation of vascular inflammation (17). Among OSA patients, chronic intermittent hypoxia induces the release of proinflammatory cytokines like IL-6, resulting in a sustained condition of mild but persistent inflammation (18).

However, for patients with moderate to severe risk for OSA with concurrent COVID-19, there is a decrease in serum IL-6, Eotaxin-1/CCL11, and salivary MIP-3α/CCL20. Eotaxin-1/CCL11 is a chemokine primarily responsible for recruiting eosinophils to inflammation sites, especially in the context of allergic reactions (19). Conversely, MIP-3α/CCL20 is a chemokine that exhibits selective binding to CCR6, a receptor predominantly found on immature dendritic cells (DC) (20). Therefore, MIP-3α/CCL20 facilitates the migration of immature DCs to the site of injury, and studies conducted by Reibman et al. have provided insight into the role of MIP-3α/CCL20 in shaping the adaptive immune response within the airway mucosa (20). The observed attenuated immune response in patients with a moderate to severe risk of OSA and concurrent SARS-CoV-2 infections may be attributed to two potential mechanisms. First, OSA-mediated chronic inflammation due to persistent intermittent hypoxia could result in immune dysfunction (21). This pre-existing immune impairment could potentially exacerbate when individuals with OSA contract SARS-CoV-2, resulting in decreased secretion of cytokines. For instance, obesity, a shared risk factor for both OSA and COVID-19, impairs the immune response through chronic low-grade inflammation, hyperinsulinemia, hyperglycemia, and hyperleptinemia (22). Additionally, the deposition of adipose tissues can impede respiratory function, increasing susceptibility to respiratory infections like COVID-19 (22). Alternatively, SARS-CoV-2 infection could directly induce an “immunological collapse,” a state of reduced inflammatory reactions, unchecked viral replication, widespread dissemination, and direct host cell cytotoxicity (23). Nonetheless, the latter theory falls short of providing a comprehensive explanation for the observed reduction in IL-6 in the current study. This is evident as Remy et al. noted an increase in IL-6 when compared to the healthy control group in their own study (23). The discrepancies between the findings further reinforces the former theory that a synergistic effect exists between OSA and the concurrent COVID-19.

### Limitations

It is important to note, however, that while the results of our cross-sectional study suggest a possible association between OSA, impaired inflammatory reaction, and increased COVID-19 severity, additional research is required to establish a definitive causal relationship. One notable limitation of this study is the relatively small sample size, which may constrain the generalizability of our findings and limit the statistical power to detect subtle associations between variables. Another limitation of our study lies in the composition of the control group, consisting of hospital staff, while the experimental group comprises hospitalized patients. The inherent differences between these groups may introduce confounding variables related to occupational exposures, health behaviors, or access to healthcare, potentially impacting the generalizability of our findings. Furthermore, the exclusive focus on hospitalized patients within our experimental groups may inadvertently disregard potential variations in cytokine profiles among non-hospitalized individuals with COVID-19, constraining the generalizability of our findings. Additionally, in the present study, polysomnography confirmation of OSA was not feasible due to the clinical status of the patients and the infection control measures implemented during the pandemic. Thus, the patients in the study were never formally diagnosed with OSA. Likewise, we did not confirm participants’ prior OSA diagnosis. This decision was influenced by the potential for introducing recall bias, and the survey’s limited length constrained the comprehensive collection of accurate historical data on past OSA diagnosis. The absence of polysomnography or stratification of participants with a prior diagnosis of OSA limits the interpretation of the results, limiting our ability to assess the impact of pre-existing OSA on the observed outcome. However, the STOP-Bang questionnaire, a validated screening tool, was employed to assess the risk for OSA (15). While the STOP-Bang questionnaire is a valuable tool, it has inherent limitations. Future investigations should explore the inclusion of prior OSA diagnoses and polysomnography confirmation. Additionally, considerations such as sample size, representative sample, controlling for additional covariates and confounding variables, and investigating potential mechanisms through which OSA may impact immune response warrant attention in future studies.

## Conclusion

Findings from the present study demonstrated an association between a moderate to high risk of OSA and the manifestation of severe COVID-19 symptoms. Moreover, the study has unveiled a link between the levels of inflammatory cytokines in individuals with OSA, some of which exhibited distinct differences based on the presence or absence of acute COVID-19. These findings substantiate the initial hypothesis, implying that individuals at higher risk of OSA may display an impaired inflammatory response, potentially intensifying the severity of COVID-19 symptoms.

Consequently, it becomes imperative to address and target OSA as a potential risk factor to mitigate the onset of severe COVID-19 symptoms in subsequent variants and respiratory infections. By identifying and implementing appropriate interventions for individuals with OSA, healthcare professionals have the potential to diminish the susceptibility to COVID-19 symptoms and the associated complications they entail. Despite the ongoing endeavors to study COVID-19, further research is imperative to attain a more comprehensive grasp of the contributing factors that exacerbate the manifestation of symptoms related to COVID-19 and other respiratory infectious diseases.

## Data availability statement

The original contributions presented in the study are included in the article/supplementary material, further inquiries can be directed to the corresponding author.

## Ethics statement

The studies involving humans were approved by Kuwait Ministry of health ethics approval: #2020/1462 Harvard: IRB21-1492, University of Alberta: Pro00125245. The studies were conducted in accordance with the local legislation and institutional requirements. The participants provided their written informed consent to participate in this study.

## Author contributions

YD: Data curation, Formal analysis, Investigation, Writing – original draft, Conceptualization. AA: Data curation, Formal analysis, Software, Conceptualization, Investigation, Writing – original draft. HesA: Conceptualization, Formal analysis, Writing – review & editing. KA: Writing – review & editing, Formal analysis, Investigation, Software. MF: Data curation, Writing – review & editing, Methodology. MA: Writing – review & editing, Conceptualization, Resources. SD: Funding acquisition, Project administration, Resources, Writing – review & editing. FA: Funding acquisition, Project administration, Resources, Writing – review & editing. SB-H: Conceptualization, Data curation, Methodology, Project administration, Supervision, Funding acquisition, Investigation, Resources, Validation, Writing – review & editing. HenA: Conceptualization, Data curation, Formal analysis, Funding acquisition, Investigation, Methodology, Project administration, Resources, Software, Supervision, Writing – original draft, Writing – review & editing.
